# Monosodium glutamate (MSG) intake is associated with the prevalence of metabolic syndrome in a rural Thai population

**DOI:** 10.1186/1743-7075-9-50

**Published:** 2012-06-08

**Authors:** Tonkla Insawang, Carlo Selmi, Ubon Cha’on, Supattra Pethlert, Puangrat Yongvanit, Premjai Areejitranusorn, Patcharee Boonsiri, Tueanjit Khampitak, Roongpet Tangrassameeprasert, Chadamas Pinitsoontorn, Vitoon Prasongwattana, M Eric Gershwin, Bruce D Hammock

**Affiliations:** 1 Department of Biochemistry, Faculty of Medicine, Khon Kaen University, Khon Kaen, 40002, Thailand; 2 Division of Rheumatology, Allergy and Clinical Immunology, University of California at Davis School of Medicine, Davis, CA, USA; 3 Clinical Immunology, Humanitas Clinical and Research Center, Milan, Italy; 4 Department of Entomology, University of California at Davis, Davis, CA, USA

**Keywords:** Functional food, Obesity, Metabolic syndrome, Thailand

## Abstract

**Background:**

Epidemiology and animal models suggest that dietary monosodium glutamate (MSG) may contribute to the onset of obesity and the metabolic syndrome.

**Methods:**

Families (n = 324) from a rural area of Thailand were selected and provided MSG as the sole source for the use in meal preparation for 10 days. Three hundred forty-nine subjects aged 35–55 years completed the study and were evaluated for energy and nutrient intake, physical activity, and tobacco smoking. The prevalence of overweight and obesity (BMI ≥ 25 kg/m^2^), insulin resistance (HOMA-IR >3), and the metabolic syndrome (ATP III criteria) were evaluated according to the daily MSG intake.

**Results:**

The prevalence of the metabolic syndrome was significantly higher in the tertile with the highest MSG intake. Further, every 1 g increase in MSG intake significantly increased the risk of having the metabolic syndrome (odds ratio 1.14, 95% confidence interval-CI- 1.12 - 1.28) or being overweight (odds ratio 1.16, 95% CI 1.04 - 1.29), independent of the total energy intake and the level of physical activity.

**Conclusion:**

Higher amounts of individual MSG consumption are associated with the risk of having the metabolic syndrome and being overweight independent of other major determinants.

## Background

The metabolic syndrome is a cluster of cardiovascular risk factors including major risk factors are abdominal obesity and insulin resistance. Moreover, other risk factors have been proposed including physical inactivity, aging, smoking, and carbohydrate and fat dietary intake [1]. The incidence and prevalence of the metabolic syndrome are rapidly growing causing a significant burden for health systems worldwide [2].

Monosodium glutamate (MSG) is a flavor enhancer largely used in the food industry with individual consumption steadily increasing worldwide. Recent cross-sectional and longitudinal studies in healthy Chinese subjects correlated MSG intake with an increased risk of being overweight irrespective of the total calorie intake and physical activity [3,4]. Animal models support a causative association between obesity and neonatal or maternal administration of high doses of MSG [5], possibly acting on immature neurological mechanisms that regulate food intake and energy expenditure [6]. Similarly, an elevated dietary MSG intake in rodent models leads to increased in serum triglyceride [7], fasting glucose [8], and insulin levels [7,8], the metabolic disorder markers.

To determine whether MSG is associated with the metabolic syndrome in humans, we conducted a study in the general adult population of a rural area of Thailand and report herein that the daily intake significantly correlates with the prevalence of the syndrome, irrespective of physical activity and calorie intake.

## Methods

### Participants

The study included families from six villages within the Khon Kaen province. The chosen villages included 880 families with a total of 4472 subjects that were screened through the involvement of health volunteers between October 2009 and April 2010. Following this first screening, we identified 324 families with 487 participants fulfilling the inclusion criteria, i.e. (i) preparing meals for family members at least two times each day, (ii) reporting the regular use of MSG as food additive, (iii) including a member between the ages of 35–55 years, and (iv) agreeing to take part in the study. Family members (n = 1–3 per family) of both sexes within the target age range were trained by health volunteer and research personnel to maintain a detailed dietary record for the duration of the study. Participants had their medical history taken including physical activity, tobacco smoking, family history of diabetes mellitus and ongoing medical treatments. We excluded participants who had chronic pulmonary, heart, kidney, liver, or psychiatric condition, established diabetes, active neoplasia or were receiving any prescription medication at the time of screening. The use of non steroidal anti-inflammatory medications or acetaminophen less than once a week was allowed. Participants were provided with 250 g of MSG in a plastic box to be used as the sole source of MSG for food preparation for 10 days. The amount utilized was registered at the end of this period (based on the returned box) and then divided by the number of subjects over 10 years of age in the household to estimate the individual use expressed as g/person/day. The study design and procedures were approved by the Human Ethical Committee of Khon Kaen University, Thailand, followed the ethical guidelines of the most recent Declaration of Helsinki (Edinburgh, 2000), and all participants gave their written consent prior to enrollment.

### Medical history

Calorie intake was calculated in randomly selected three-day periods in different villages using a 24-hour recall diary for which participants had been previously trained by health volunteers. All foods, drinks, and supplements were included using cups and spoons as units for clarity purposes. Diet data were coded and analyzed using the INMUCAL program version 7, software package developed by the Institute of Nutrition, Mahidol University, to determine daily energy and nutrient intakes [9]. This software provides an accurate measurement of the dietary intake of participants as part of each meal and subdivided into energy (Kcal), carbohydrate (g), lipid (g) and protein (g) contents to be used for statistical analysis. Participants were asked to indicate their levels of physical activity which was arrayed into sedentary, moderately active, and vigorously active. The modified version of the International Physical Activity Questionnaire (IPAQ) [10] was administered at a random date of the 10-day MSG supplementation period. The questionnaire is divided into 3 items assessing (i) the physical activity related to household chores such as cooking, cleaning and gardening, (ii) exercise and sports, and (iii) work-related activity. The physical activity levels (PAL) were subsequently calculated according to the 2001 FAO/WHO/UNU expert consultation [11]. All participants were asked about tobacco smoking and family history of diabetes as potentially confounding factors.

### Physical examination and bloodwork

Participants who completed MSG assessment and lifestyle covariates underwent a physical examination and blood collection for biochemical analyses. Blood pressure was measured in the sitting position after resting for a few minutes using an automatic sphygmomanometer. The waist circumference was measured at a level midway between the lower rib margin and the iliac crest while the participants stood. Body weight was measured in light indoor clothing without shoes and height measured using a secured ruler; the body mass index (BMI) was calculated accordingly. Following overnight fasting, a venous blood sample was obtained to determine the concentrations of triglyceride, total and HDL cholesterol, glucose, and insulin using routine laboratory methods. The Homeostasis Model Assessment for insulin resistance (HOMA-IR) was calculated using the formula: [fasting serum insulin (μU/ml) × fasting plasma glucose (mmol/L)]/22.5 [12]. Insulin resistance was defined as HOMA-IR >3. Overweight was defined as BMI ≥25 kg/m^2^ according to the WHO criteria [13]. The diagnosis of metabolic syndrome was made according to the National Cholesterol Education Program Adult Treatment Panel III (ATP III) (2004) criteria [1]. Three or more of the five following criteria characterized the metabolic syndrome: abdominal obesity, waist circumference ≥90 cm for men and ≥80 cm for women; systolic blood pressure ≥130 mmHg and diastolic blood pressure ≥85 mmHg; fasting plasma glucose >100 mg/dL; serum triglyceride ≥150 mg/dL; HDL cholesterol <40 mg/dL) for men and <50 mg/dL for women.

### Statistical analysis

For the purpose of statistical analysis, participants were subdivided into tertiles of MSG intake expressed as median (interquartile range, IQR). Baseline characteristics were first assessed according to tertiles of MSG consumption. Continuous variables are reported as medians and IQR (i.e., values corresponding to the 25^th^ and the 75^th^ percentiles). Differences between tertiles were tested for statistical significance by chi-square test for categorical variables and by Kruskal-Wallis test for continuous variables. The Mantel-Haenszel chi-square test was utilized to examine the linear trend of prevalence of insulin resistance, overweight, and metabolic syndrome, and its components across tertiles of MSG intake. The association of MSG consumption with metabolic syndrome was assessed by binary logistic regression analysis adjusting for predefined sets of independent variables such as sex, age, family history of diabetes mellitus, smoking status, physical activity levels and total energy intake. All analyses were two-tailed and performed using SPSS 16.0 for Windows (IBM Company, Chicago, IL). P values <0.05 were considered as statistically significant.

## Results

We obtained complete information from 349/487 (72%) participants. The remaining 138 (28%) participants dropped out of the study by not attending physical examination and blood collection (n = 113), providing insufficient questionnaire data (n = 11) and/or blood amount (n = 13). An average MSG intake of 4.0 ± 2.2 (range 0.4–14.0) g/day was recorded with a distribution skewed towards lower values. When divided into 3 groups, the median MSG intake for each tertile was 1.9 g (IQR 1.5–2.3) for tertile 1, 3.6 g (IQR 3.2–4.2) for tertile 2, and 6.0 g (IQR 5.2–7.4) g/day for tertile 3 (Table 1). There were no significant differences in sex distribution, family history of diabetes, smoking status, physical activity, and daily energy intake among participants in the different tertiles, with the exception of age (P = 0.044). The majority of participants was classified as non-smokers (256/349, 73%) and had a vigorously active lifestyle (231/349, 66%). Insulin resistance was diagnosed in 96/349, 28% of participants with 130/349, 37% were classified as overweight, and 83/349, 24% fulfilling the metabolic syndrome diagnostic criteria. When single criteria of metabolic syndrome were analyzed, the overall prevalence of enhanced waist circumference was 124/349, 36%, hyperglycemia 142/349, 41%, hypertriglyceridemia 145/349, 42%, low HDL cholesterol 112/349, 32%, and hypertension 77/349, 22%. There were no differences in levels of fasting blood glucose, triglycerides, HDL cholesterol, blood pressure, waist circumference, and BMI among tertiles of MSG intake, with the exception of insulin plasma levels (P = 0.041). A significant trend towards increasing prevalence across the different tertiles of MSG intake was observed for the prevalence of insulin resistance (P = 0.028), overweight (P = 0.021), and metabolic syndrome (P = 0.014). The prevalence of hyperglycemia, elevated blood pressure, and enhanced waist circumference apparently increased towards the highest tertile of MSG intake but differences did not reach a statistical significance.

**Table 1:** Main participant characteristics according to Tertiles of MSG intake

**Characteristics**	** Total (n = 349)**	**MSG intake**			***P***
		**Tertile 1 (n = 116)**	**Tertile 2 (n = 117)**	**Tertile 3 (n = 116)**	
**Median MSG intake (g/day)**	3.6 (2.3–5.25)	1.9 (1.5–2.3)	3.6 (3.2–4.2)	6.0 (5.2–7.4)	
**Women**	217 (62.2%)	72 (62.1%)	72 (61.5%)	73 (62.9%)	0.976 ^2^
**Age (years)**	46.0 (40.0–50.0)	44.0 (40.0–49.0) ^1^	46.0 (40.0–51.0)	47.0 (42.0–50.0)	0.044 ^2^
**Family history of diabetes**	102 (29.2%)	36 (31.0%)	31 (26.5%)	35 (30.2%)	0.721 ^2^
**Smoking status**					
Never smoked	256 (73.3%)	84 (72.4%)	83 (71.0%)	89 (76.7%)	0.264 ^2^
Former	10 (2.9%)	3 (2.6%)	4 (3.4%)	3 (2.6%)	
Current	83 (23.8%)	29 (25.0%)	30 (25.6%)	24 (20.7%)	
**Physical activity/lifestyle**					
Sedentary	48 (13.7%)	14 (12.1%)	17 (14.5%)	17 (14.7%)	0.721 ^2^
Moderately active	70 (20.1%)	27 (23.3%)	19 (16.3%)	24 (20.7%)	
Vigorously active	231 (66.2%)	75 (64.6%)	81 (69.2%)	75 (64.6%)	
**Daily nutrient intake**					
Energy intake (Kcal)	2,032 (1,747–2,397)	2,051 (1,758–2,381) ^1^	2,029 (1,687–2,380)	2,017 (1,765–2,413)	0.905 ^2^
Carbohydrate (g)	333.8 (275.8–412.3)	342.2 (280.9–413.8) ^1^	328.1 (260.2–422.7)	341.2 (280.8–407.2)	0.569 ^2^
Fat (g)	33.1 (25.9–43.4)	34.1 (26.1–42.1) ^1^	32.8 (26.3–44.2)	31.7 (24.5–43.7)	0.739 ^2^
Total protein (g)	81.1 (68.8–96.9)	82.5 (70.7–97.6) ^1^	81.1 (67.6–93.0)	70.4 (67.5–95.3)	0.437 ^2^
**Metabolic parameters**					
HOMA-IR	2.3 (1.7–3.3)	2.1 (1.6–2.8) ^1^	2.3 (1.6–3.3)	2.4 (1.9–3.5)	0.028 ^2^
% HOMA-IR >3	96 (27.5%)	24 (20.7%)	33 (28.2%)	39 (33.6%)	0.028 ^3^
Insulin (ng/dL)	9.1 (7.0–13.2)	8.5 (6.6–12.0) ^1^	9.0 (6.8–13.0)	9.9 (7.5–14.1)	0.041 ^2^
Glucose (mg/dL)	97.0 (92.0–104.0)	97.0 (92.0–104.0) ^1^	98.0 (91.5–103.5)	99.0 (92.0–105.0)	0.647 ^2^
% glucose >100 mg/dL	142 (40.7%)	40 (30.7%)	48 (40.9%)	54 (43.0%)	0.062 ^3^
Triglyceride (mg/dL)	137.7 (104.1–191.1)	139.1 (95.1–197.4) ^1^	139.0 (104.0–203.1)	134.2 (109.7–187.8)	0.729 ^2^
% Triglycerides ≥150 mg/dL	145 (41.5%)	48 (41.4%)	49 (41.9%)	48 (41.4%)	0.996 ^3^
HDL-C (mg/dL)	53.0 (44.0–63.0)	51.0 (41.0–62.8) ^1^	53.0 (45.0–65.0)	53.5 (45.0–64.0)	0.407 ^2^
% HDL-C <50 mg/dL F, <40 mg/dL M	112 (32.1%)	44 (37.9%)	34 (29.1%)	34 (29.3%)	0.160 ^3^
SBP (mmHg)	124.0 (116.0–136.0)	124.0 (116.0–133.8) ^1^	125.0 (116.0–135.5)	123.0 (115.3–136.8)	0.910 ^2^
DBP (mmHg)	79.0 (72.5–86.0)	78.0 (73.0–85.0) ^1^	80.0 (75.0–86.0)	80.0 (71.3–87.0)	0.503 ^2^
% SBP ≥ 130 and DBP ≥ 85 mmHg	77 (22.1%)	21 (18.1%)	27 (23.1%)	29 (25.0%)	0.206 ^3^
Waist circumference (cm)	79.0 (72.0–86.0)	78.5 (72.6–85.0) ^1^	77.0 (71.9–86.0)	81.0 (73.0–88.0)	0.207 ^2^
%WC >80 cm F, >90 cm M	124 (35.5%)	39 (33.6%)	35 (29.9%)	50 (43.1%)	0.132 ^3^
BMI (kg/m^2^)	23.7 (21.2–26.8)	23.5 (21.3–26.4) ^1^	23.1 (20.9–26.8)	24.6 (21.3–27.3)	0.308 ^2^
% BMI ≥ 25 kg/m^2^	130 (37.2%)	36 (31.0%)	41 (35.0%)	53 (45.7%)	0.021 ^3^
**Metabolic syndrome (ATP III criteria)**	83 (23.8%)	20 (17.2%)	27 (23.1%)	36 (31.0%)	0.014 ^3^

^1^ Continuous variables are expressed as median (interquartile range).

^2^*P* value comparing the characteristics of participants across tertiles of MSG intake using chi-square test for categorical variables and by Kruskal-Wallis test for continuous variables.

^3^*P* values comparing prevalence of each metabolic feature across tertiles of MSG intake using the Mantel-Haenszel chi-square test for trend.

*HDL-C* high density lipoprotein cholesterol, *F* female, *M* male, *SBP* systolic blood pressure *DBP* diastolic blood pressure, *WC* Waist circumference, *BMI* body mass index.

The binary logistic regression analysis with potentially confounding factors is illustrated in Table 2. MSG intake was independently associated with having the metabolic syndrome after adjustment for potential confounding factors such as calories intake, age, sex, physical activity level, smoking level, and history of diabetes. Further, every 1-g increase in MSG intake slightly but significantly increased the odds of metabolic syndrome (odds ratio 1.14, 95% CI 1.12–1.28) and overweight (odds ratio 1.16, 95% CI 1.04–1.29) increased significantly.

**Table 2:** Odds ratio and 95% CI for MSG intake associated with increased risks of insulin resistance, overweight, and metabolic syndrome

	**Number of participants**	**OR (95%CI)**	***P***
**Insulin resistance (HOMA-IR > 3)**	349	1.05(0.94–1.18)*	0.371
**Overweight (BMI ≥ 25 kg/m**^**2**^**)**	349	1.16(1.04–1.29)*	0.007
**Metabolic syndrome (ATPIII criteria)**	349	1.14(1.12–1.28)*	0.025

* adjusted for sex, age, physical activity level, smoking level, history of diabetes and calories intake.

## Discussion

The present study reports a significant association between the risk of the metabolic syndrome and MSG daily consumption in a rural area of Thailand where people cook their own meals with the typical eating habit of sharing food among family members. Importantly, the association is independent of major confounding factors such as age, sex, smoking, family history, physical activity, or calorie intake. This unique context allowed the measurement of MSG consumption for 10 days to be more accurate than the use of questionnaires.

The metabolic syndrome is currently considered as the most prominent risk factor for cardiovascular diseases worldwide. Data from the Third National Health and Nutrition Examination Survey (NHANES III) on 8,814 men and women suggest that 20 to 30% of adults in developed countries meet the syndrome criteria [14]. In addition to an elevated calorie intake [15], specific dietary factors have been linked with the incidence of the syndrome with conflicting results [16]. MSG is a common flavor enhancer and naturally occurring amino acid utilized in increasing amounts in food industry and preparation. Despite its optimal safety profile [17], a large cross-sectional study revealed that MSG consumption is related to a higher increase in blood pressure [18]. Moreover, MSG has been linked with obesity, type II diabetes, and the metabolic syndrome as its intake in healthy Chinese adults correlates with the resulting increase in body mass index regardless of energy intake [3,4].

We found a significant trend for increasing insulin levels and prevalence of insulin resistance across tertiles of MSG intake. However, the change did not alter the glucose homeostasis significantly as fasting blood sugar did not differ among different levels of MSG intake. This is in agreement with the observation that the administration of MSG to fasting human subjects increases insulin levels without altering glucose concentration and glucose tolerance [19]. While glutamate is known to stimulate insulin secretion via glutamate receptor, the optimal glucose regulation in healthy volunteer may overcome the intermittent rising of insulin level [19,20].

Further evidence of a link between MSG and the metabolic disorders is obtained from animal models. High doses of MSG injected in rodents during the neonatal period lead to the development of glucose intolerance, insulin resistance [21], and obesity [5] along with adipose tissue hypertrophy, hyperinsulinemia, hyperglycemia, hyperleptinemia, and decreased insulin stimulated glucose transport in adipocytes and muscle [22-24]. These changes in glucose metabolism and insulin resistance were not observed when MSG was administered to adult animals, thus suggesting that the endocrine regulation in these animals is very sensitive to MSG during the early postnatal period [23]. The metabolic disturbances found in this present study might be the sum of exposure during early life via the maternal diet including in their later life [25].

MSG is a common additive used in Thai cuisine and we observed an average MSG intake of 4.0 g/day which was over two times higher than an older survey of Thai adults [26]. The MSG intake in this study was similar to that recent report in Chinese population (3.8 g/day) by the Jiangsu Nutrition Study [27]. However, our recorded MSG intake was 12 and 2 times higher compared to that of the two Chinese population nutritional surveys, i.e. the INTERMAP study [3] and the China Health and Nutrition Survey (CHNS) [4], respectively. While we can only speculate on the impact of different eating habits, our measurement of MSG consumption based on common cooking habits in a limited area with uniform dietary and physical activity regimens. Further, to be included in the present study families had to prepare their meals twice a day thus limiting the impact of commercially available food. Lastly, inhabitants of Thai rural areas rarely eat restaurant food compared to urban areas, thus minimizing this potential confounding factor.

Additional observations strengthen our hypothesis. First, we should note that daily energy intake and physical activity levels are not significantly different among tertiles of MSG consumption, possibly based on the fact that 66% of participants are agricultural workers with a vigorously active lifestyle. Second, the prevalence rates of hypertriglyceridemia and low HDL cholesterol levels are not associated with MSG intake, while the overall prevalence of hypertriglyceridemia is similar to data from Thai adults in rural areas [28]. Our data demonstrate the significant trends of insulin resistance, overweight, and metabolic syndrome towards levels of MSG intake. However, after adjusting for the confounding factors such as sex, age, calories intake, physical activity, smoking status, and history of diabetes, the MSG consumption does not significantly increase risk of insulin resistance. Since there were no differences in glucose level and energy intake in different tertiles of MSG consumption, the metabolic syndrome associated with MSG intake is probably not due to the energy intake-induced obesity leading to insulin resistance. The finding of MSG consumption significantly correlated with BMI in our study agreed with the two studies in Chinese population, INTERMAP and CHNS [3,4], through a mechanism possibly mediated by white adipose tissue deposition, as suggested in animal models [7,23,29]. MSG may enhance shifting the dietary glucose towards lipid synthesis [23], increasing the rate of lipogenesis [29] and activating gene expression of enzymes involved in lipid biosynthesis and storage in adipose tissue [7].

Our results demonstrate that MSG intake is associated with the risk of having the metabolic syndrome. A longitudinal study with a larger sample size is needed for confirmation the contribution effect of MSG on the development of metabolic syndrome, a rising global metabolic emergency.

## Conclusions

The present study supports that an elevated dietary MSG consumption is significantly associated with having the metabolic syndrome and being overweight in a Thai rural population. We may surmise that a person with a daily consumption of MSG exceeding 5 g should be considered at risk for metabolic disorder.

## Competing interests

The authors declare no competing interest.

## Authors’ contributions

The project idea for this study came from UC. TI and UC had full access to all the data, were responsible for literature review and the first draft of the paper. TI, UC and SP analyzed and interpreted the data. CS, UC are responsible for the literature review and manuscript preparation. PY, PA, PB, TC, RT, CP, VP are responsible for data and blood collection. MEG and BDH provided major suggestions during the study and the manuscript preparation. All authors have read and approved the final manuscript.
